# Supplementary material for: An Off-the-Shelf Approach for the Production of Fc Fusion Proteins by Protein Trans-Splicing towards Generating a Lectibody In Vitro
Source: Int J Mol Sci. 2020 Jun 3;21(11):4011. doi: 10.3390/ijms21114011 (PMC7313076; doi:10.3390/ijms21114011)
Supplement: Supplementary file 1 [file ijms-21-04011-s001.pdf]

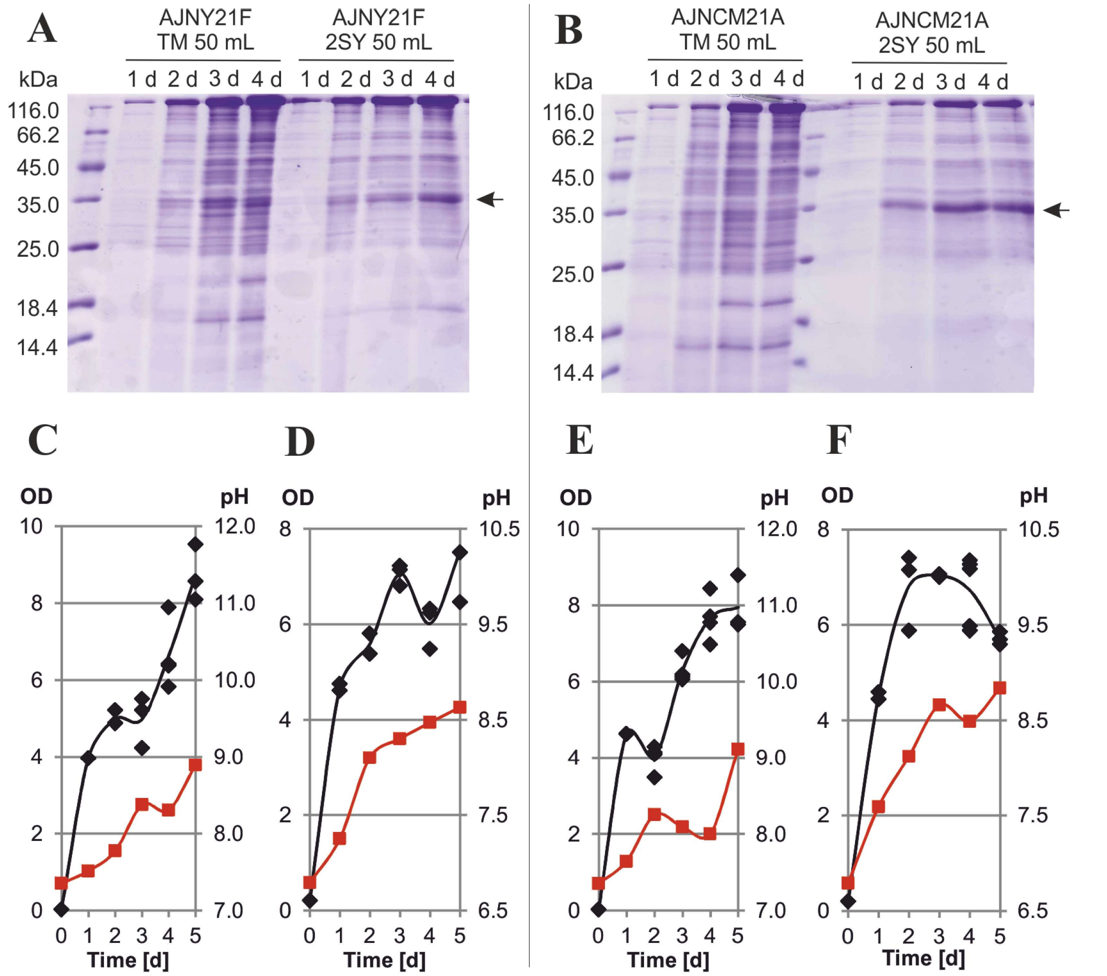

Supplemental Fig. S1: Comparison of protein expression of Int$_C$-CPPC-Fc with pAJNY21 with P5 promoter (A,C,D) and pAJNCM21A with P2 promoter (B,E,F). **(A, B)** SDS-PAGE analysis of the culture supernatant during four days. (**C,D,E,F**) Optical density at 600 nm and pH of the cell culture during five days.

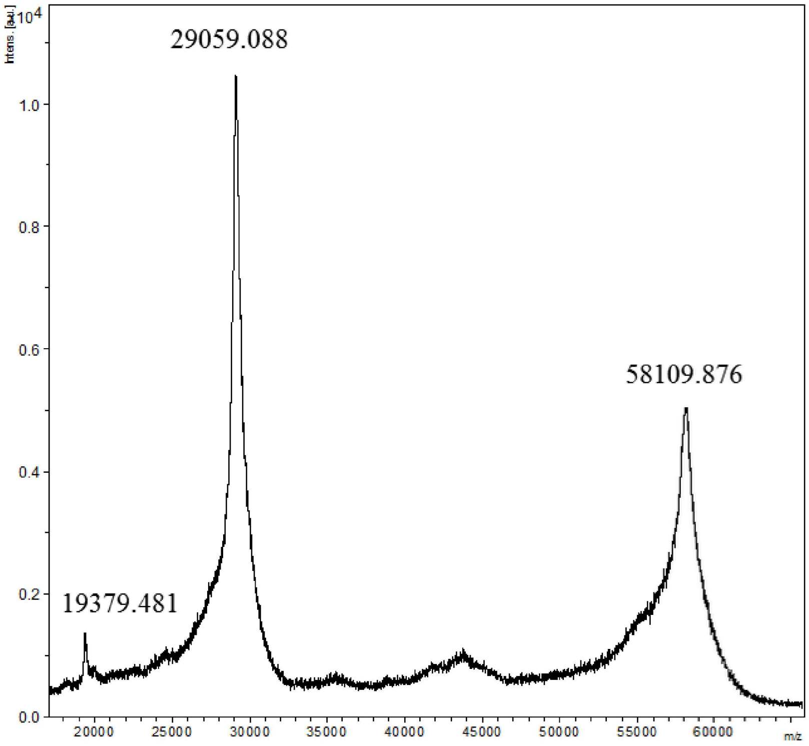

Supplemental Fig. S2: Molecular mass analysis of IntC-CPPC-Fc by MALDI-TOF spectrometry. The calculated molecular mass for the monomeric Int_C-CPPC-Fc (AJNCM21A) is 29057.8 Da. The dimer is expected to have 58115.6 Da.

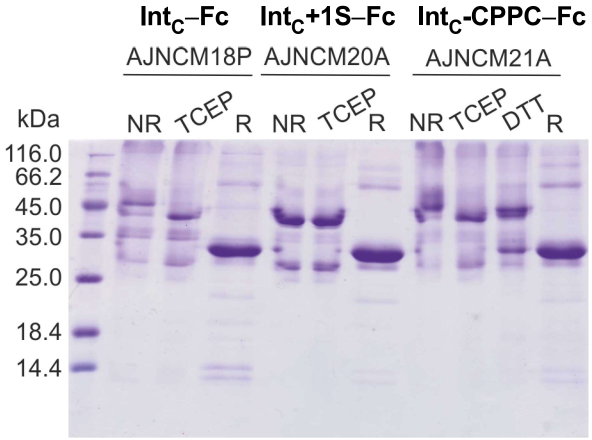

Supplemental Fig. S3: SDS–PAGE analysis the three Fc constructs of reducing conditions Samples were either prepared in an excessively reducing (R), non-reducing (NR) conditions. TCEP and DTT indicate the presence of 0.5 mM TCEP (TCEP) or 5 mM DTT (DTT), respectively.

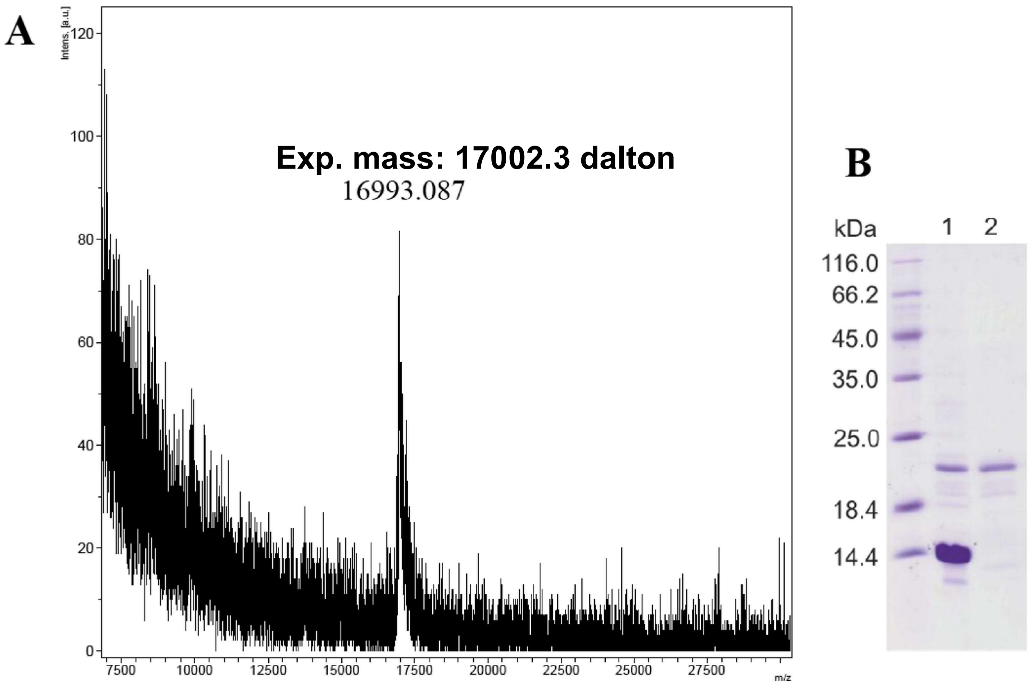

Supplemental Fig. S4: Molecular mass analysis of SVN-GB1. (A) MALDI-TOF spectrum of the purified fraction of SVN-GB1**. (B) SDS-PAGE analysis of the reaction mixture.** The reaction mixture (B, lane 1) was purified by reversed-phase chromatography. The lane 2 in the SDS-gel was anlalyzed by MALDI-TOF mass spectrometry. The expected mass was calculated for [^{15}N]-SVN-[^{14}N]-GB1.
